# Petrophysical characterization and chemical treatment of oil reservoir as a tool for choosing the best improved oil recovery techniques

**DOI:** 10.1038/s41598-026-56320-z

**Published:** 2026-06-24

**Authors:** Samah A. M. Abou-alfitooh, Mohamed H. Farag, Fatma M. Abdelhafiz, Nashwa M. Saleh, Nour E. A. Abd El-Sattar, Rasha S. Kamal

**Affiliations:** 1 Enhanced Oil Recovery Laboratory, Production Department, Egyptian Petroleum Research Institute (EPRI), Nasr City, Cairo, Egypt; 2 Geophysics Laboratory, Exploration Department, Egyptian Petroleum Research Institute (EPRI), Nasr City, Cairo, Egypt; 3 EPRI Core Analysis Center, Egyptian Petroleum Research Institute (EPRI), Nasr City, Cairo, Egypt; 4 Petrochemicals Department, Egyptian Petroleum Research Institute, Nasr City, Cairo, 11727 Egypt; 5 Department of Chemistry, Faculty of Science, Al‐Azhar University (Girls Branch), PO Box 11754, Cairo, Egypt; 6 Department of Chemistry, Faculty of Science, Ain Shams University, Abbassia, Cairo, Egypt; 7 Basic and Medical Sciences Department, Faculty of Dentistry, Alryada University for Science and Technology, Sadat City, Egypt; 8 Department of Petroleum Applications, Egyptian Petroleum Research Institute, Nasr City, Cairo, Egypt

**Keywords:** Core chemical flooding, Rock wettability, Interfacial tension, Crude oil, Chemistry, Energy science and technology, Engineering, Environmental sciences

## Abstract

Enhanced oil recovery (EOR) methods are essential for maximizing oil extraction from mature reservoirs. Given the ongoing reliance on crude oil, it is essential to advance enhanced oil recovery techniques to boost reservoir production and extend their lifespan. Among the chemical EOR methods, chemical flooding is a well-established technique that can theoretically be utilized across various reservoir conditions. In this paper three novel bis (ethanethioyl) oxalamide derivatives synthesized via an eco-friendly green chemistry route using ethanol solvent at ambient temperature as chemical flooding agents. Their molecular efficacy was rationalized by quantum chemical (DFT) calculations and FTIR spectroscopy, which linked optimal interfacial activity to specific structural features. They were tested as an agent in reducing the interfacial tension (IFT) between the injected water and crude oil and also, in altering the wettability of reservoir rock. The results indicated the efficiency of the new compound (bis N) in reducing the IFT from 27 to 5 mN/m also altering the rock’s affinity for water than oil. Finally, this agent was used in chemical flooding experiments on real core plugs under reservoir conditions in terms of (temperature, pressure, and crude oil). From flooding experiments, these calculations indicate positive economics for enhanced oil recovery through this new compound where it can withstand severe reservoir conditions and achieve a recovery factor of 22.82%S_or_, 29.75%S_or_ and 34.12%S_or_ in the case of 1 g/l, 1.5 g/l and 2 g/l concentrations respectively, from the remaining oil.

## Introduction

The global energy landscape is currently facing a critical transition as the discovery of new giant oil fields becomes increasingly rare, and production from existing conventional reservoirs continues to decline^1–5^. Most of the world’s major hydrocarbon assets have reached a “mature” or “declining” stage. In these reservoirs, natural drive mechanisms such as gas cap expansion or solution gas drive have been exhausted, and secondary recovery methods like water flooding are no longer capable of maintaining economic production rates^6–10^. Statistical data indicate that after traditional recovery stages, a substantial volume of original oil in place (OOIP) often exceeding 60% remains trapped in the reservoir as residual oil^11–15^. The causes of residual oil and suboptimal recovery rates are many^16,17^, encompassing inadequate petrophysical characteristics, diminutive pore throats, considerable variability, low oil saturation, the existence of clay minerals, and adverse wettability. Usually, flow resistance is very high, which makes it hard to displace the oil and difficult to inject water^18–20^. It is crucial to highlight the usage of further enhanced oil recovery (EOR) techniques while talking about the importance of EOR^21–24^ and the present status of hydrocarbon development across different nations and major oil and gas fields. Advanced technologies used in tertiary enhanced oil recovery (EOR) techniques change the reservoir fluids’ initial characteristics^25,26^. Although they can be used at any time during the reservoir’s existence, they are usually used in the last phase of field development. Chemical, thermal, and gas injection techniques are the three most used forms of tertiary EOR^27–30^. A number of variables, such as reservoir temperature, pressure, depth, permeability, residual oil and water saturation, porosity, and the characteristics of the formation fluids, such as density, viscosity, and salinity, influence the best utilization of each kind^31,32^. Compared to traditional enhanced oil recovery (EOR) approaches, tertiary EOR methods require more science and have more sophisticated technology. Energy is needed to raise the crude oil from the reservoir to the surface during the oil production process^33^. The majority of the energy required for production is first provided by the pressure in the natural reservoir. But as time passes and this pressure drops, more tools are needed to make extraction easier. To increase reservoir pressure, more oil can be displaced by injecting gas or water^34,35^. There is frequently a significant amount of oil left in the reservoir even after using these techniques. Tertiary EOR strategies categorized into thermal, gas, and chemical methods are specifically designed to target the immobile residual oil by altering the physical and chemical interactions at the fluid–fluid and fluid-rock interfaces^36^. Among these, Chemical Enhanced Oil Recovery (cEOR) is particularly versatile. It employs specialized agents such as surfactants, polymers, and alkalis to perform two primary functions: reducing the interfacial tension (IFT) between the oil and the displacing aqueous phase to near-zero values, and improving the mobility ratio by increasing the viscosity of the injected water. This dual action significantly enhances both the microscopic displacement efficiency and the macroscopic sweep efficiency of the flood^37^. In recent years, the focus of cEOR research has shifted toward sustainability and economic efficiency. The petroleum industry is under increasing pressure to adopt “Green Chemistry” principles, which emphasize the synthesis of high-performance surfactants using renewable materials, energy-efficient processes, and high atom economy. These sustainable routes are crucial for reducing the environmental footprint of oilfield operations and lowering the high capital expenditures associated with chemical flooding projects. However, a significant challenge remains: many conventional surfactants lose their effectiveness or degrade under the harsh conditions of high-temperature and high-salinity (HTHS) reservoirs. To address these challenges, this study explores explores the synthesis and performance of three novel organic derivatives based on oxalyl chloride and ammonium thiocyanate^38,39^. These compounds were designed to offer superior thermal stability and high surface activity. This research provides a comprehensive evaluation of these chemicals by integrating petrophysical analysis of real core plugs with advanced interfacial studies. Furthermore, the study utilizes Quantum Chemical Parameters via Density Functional Theory (DFT) to provide a molecular-level understanding of how these compounds behave at the interface. By correlating these theoretical insights with real core-flooding results under actual reservoir conditions of temperature and pressure, this work aims to provide a robust framework for selecting the most effective chemical treatments for mature oil reservoirs.

## Lab-experiments

### Synthetic procedure and confirmation the compounds

**Scheme 1:**
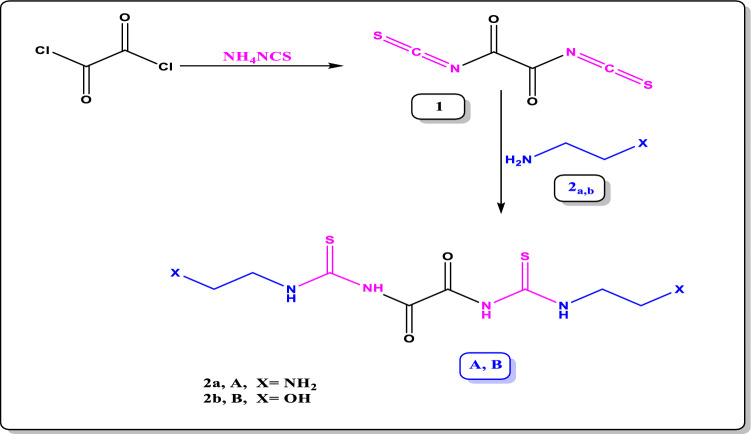
Preparation of compounds A [N^1^,N^2^-bis((2-aminoethyl) carbamothioyl) oxalamide] and B [N^1^,N^2^-bis((2-hydroxyethyl) carbamothioyl) oxalamide].

**Scheme 2:**
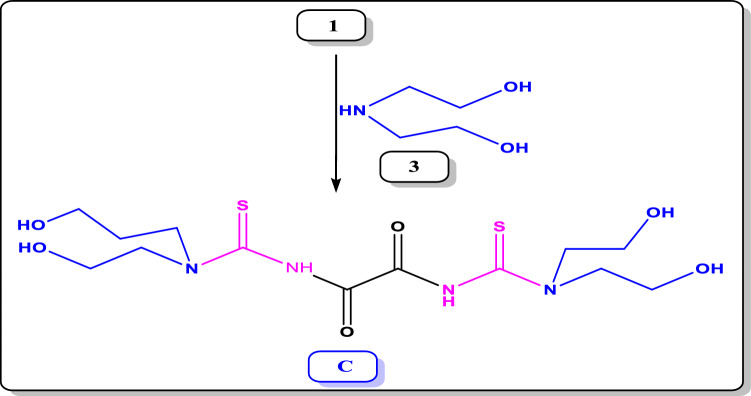
Preparation of compound C [N^1^-(bis(2-hydroxyethyl)carbamothioyl)-N^2^-((2 hydroxyethyl)(3-hydroxypropyl)carbamothioyl)oxalamide].

Three Organic Compounds (bis N, bis 1, and bis 2) were prepared in by stirring of oxalyl chloride (100 mmol) with ammonium thiocyanate (100 mmol) in dry acetone for 30 min at controlled temperature of 25 °C then filter to remove ammonium chloride after that we add different amines as ethylene diamine, ethanol amine and diethanol amine and continue stirring for another 20 min then we remove acetone to obtain the products^38,39^ as shown in Schemes 1 and 2. The structures were confirmed using FT-IR^40–45^ and study also Quantum chemical parameters^46^.

### Surface tension evaluation

The surface parameters of compounds Ia (bis N), Ib (bis 2), and Ic (bis 1) were determined by measuring the surface tension (γ) of each compound in distilled water. Three samples of each concentration within the concentration range of 0.000001–0.1 M/L were prepared and the solutions were stabilized for 10 min prior to the measurement. The average measurements for each concentration used for calculating the surface parameters.

The CMC values of the compounds under investigation were detected via surface tension technique using Attention Theta Optical Tensiometer (Biolin Scientific Company, Finland). The CMC values of the synthesized compounds at 25 °C were determined from the inflection (γ) against logarithm of the compound concentration [log C] curve. The effectiveness (π_cmc_) of the synthesized compounds was established according to Eq. (1). It corresponded to the difference in the (γ) values of distilled water (γ_o_) without the synthesized compounds and that with Ia, Ib, and Ic at their corresponding CMC (γ_CMC_)^47^:

1 $$\pi_{{{\mathrm{cmc}}}} = \gamma_{{\mathrm{o}}} - \gamma_{{{\mathrm{cmc}}}}$$

The maximum surface excess (Γ_max_) corresponded to the highest concentration at the solution interface per unit area, which could be achieved by each compound. Γ_max_ could be calculated according to the following Gibbs adsorption Eq. (2)^48^:

2 $$\Gamma_{\max } = - (\updelta \gamma /\updelta \log {\mathrm{c}})_{{\mathrm{T}}} /2.303\;{\mathrm{n}}\;{\mathrm{RT}}$$

where n for each synthesized compound equals 2, (δ*γ*/δ log c) indicates the surface pressure, while R and T refer to the gas constant and absolute temperature, respectively.

The minimum surface areas (A_min_) of the synthesized compounds Ia, Ib, and Ic were calculated according to the Gibbs adsorption (Eq. 3)^49^:

3 $${\mathrm{A}}_{\min } = 10^{16} /{\mathrm{N}}_{{\mathrm{A}}} \cdot \Gamma_{\max }$$

where A_min_ refers to the average area occupied by each compound at the interface and N indicates the Avogadro’s number.

Surface tension measurements were used to calculate the micellization and adsorption free energy at the interfaces. The adsorption of amphiphile molecule at the interface under equilibrium conditions reduces surface tension. The number of molecules adsorbed at the interface per unit area was provided by Gibbs adsorption equation. The CMC values play a vital role in calculating ΔG_mic_. Standard free energies of micellization and adsorption (ΔG^o^_mic_, ΔG^o^_ads_) were calculated using Gibbs adsorption rules^50^ as follows:

4 $$\Delta {\mathrm{G}}_{{{\mathrm{mic}}}}^{{\mathrm{o}}} = {\text{n RT ln CMC}}$$

5 $$\Delta \mathrm{G}_{{{\mathrm{ads}}}}^{{\mathrm{o}}} = \Delta \mathrm{G}_{{{\mathrm{mic}}}}^{{\mathrm{o}}} - 6.023 \times 10^{ - 1} \times \pi_{{{\mathrm{cmc}}}} \times {\mathrm{A}}_{\min }$$

where R is the gas constant, T is the absolute temperature.

### Core materials

The three sandstone core samples # 1, # 2 and # 3 of size 1.5-inch diameters were used in the core flooding experiment to test different concentrations of synthesized organic solution. Before testing, the core plugs were handled using Soxhlet extraction with toluene solvent to remove the oil and then with methanol to remove any salts. The cores were dried using a drying oven. The cores were weighted, where the bulk volume (B_V_) was calculated using a digital Vernier caliper, and then the grain volume (GV) was calculated through a helium porosimeter instrument, hence the pore volume (PV), the porosity (Ø), grain density and air permeability (Ka) were calculated (Table 1).

**Table 1:** The petro physical properties of the tested core plug samples.

Sample No	L	D	BV	PV	Porosity (%)	Grain density	Ka (mD)
# 1	5.38	3.841	62.34	15.77	25.30	2.63	152.29
# 2	5.295	3.842	61.39	16.02	26.10	2.61	191.92
# 3	5.333	3.84	61.76	15.53	25.14	2.62	131.53

Where L: Sample length, D: Sample diameter, PV: Pore volume, BV: Bulk volume, ka: Gas permeability (mD).

### Wettability test

Prior to the flooding test, the Amott wettability method was assessed^51^ to help interpret the results, where the clean and dry tested samples were evacuated and 100% saturated with the formation water. Each sample was placed in a stainless-steel core holder and flushed to immobile water saturation using mineral oil. Following this, the sample was submerged under the formation water and the volume of brine imbibed under static conditions was noted. The sample was next flooded with the brine and the volume of water imbibed dynamically recorded. The wettability index to water was calculated then. The sample was then placed under oil and the volume of oil imbibed under static conditions was determined. Once equilibrium had been reached, the sample was flooded with mineral oil. Dynamic volumes of oil imbibed were recorded and the wettability index to oil was calculated. The wettability of samples was evaluated using wettability indices for water and oil.

### Flooding test

The cores were vacuumed and then saturated with a brine solution of salinity (9.5% wt.) under the pressure of 2000 psi and left for one day to attain complete saturation. After saturation, the cores were weighed again, to ensure 100% saturation with brine. The stainless-steel core holder was used to accommodate the fully saturated core samples, where a temperature of 60 °C and a confining pressure of 2700 psi were applied. The porosity of the tested samples at reservoir conditions was determined using the pipette method where a graduated pipette was connected to the end of the core holder, where this pipette contained a certain volume of formation water as a zero level. The confining stress was then applied to the required pressure value and the reduction in the pore volume was monitored through an increase in the volume of formation water within the pipette. The difference between the new volume and zero level was recorded as the reduction in the pore volume as a result of applying overburden pressure. This reduction was subtracted from the original pore volume to get the new pore volume at the applied value of the overburden pressure, hence the new porosity was determined. The original oil in place (OOIP) was evaluated via crude oil flooding of a fully brine-saturated core sample. The oil permeability at the residual water saturation was recorded as the base permeability. To assess the residual oil saturation, the secondary flooding using formation water was carried out up to the stage of no more oil displaced out of the tested sample, where water permeability at the residual oil saturation was measured after achieving this stage. The water/oil relative permeability was traced while flooding using the method of (JBN 1959)^52^, where the remaining oil (% pv) is treated chemically. Finally, plug samples were flooded chemically using different concentrations of surfactant, where varied additional oil recoveries were recorded. All the permeability values were calculated using Darcy’s Law, where the flow rates, differential pressures, sample dimensions, and fluid viscosities were involved in the calculations.

## Results and discussion

### Characterization of synthesized compounds

The synthesized compounds were characterized using FTIR and study also Quantum chemical parameters.

**Fig. 1:**
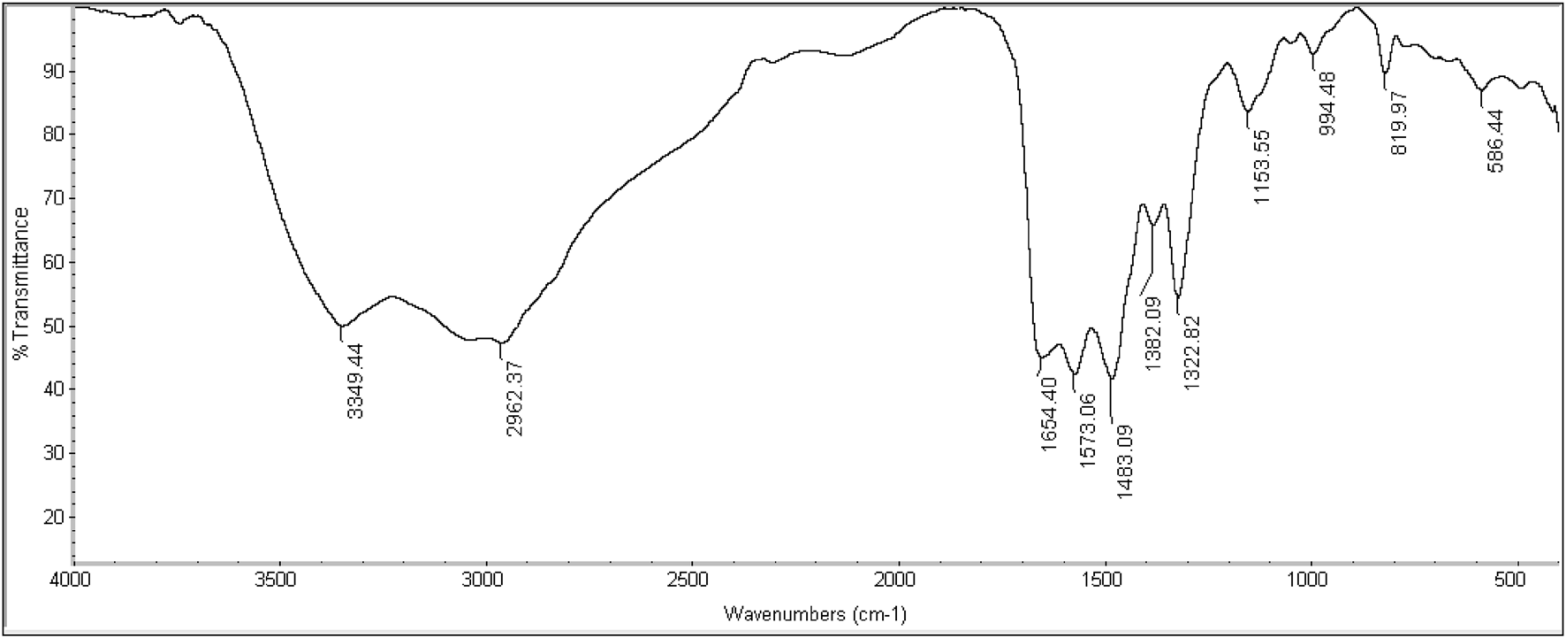
Infrared structure of the prepared compound (A).

Figure 1 is shown the FTIR spectrum of compound (A) exhibits characteristic absorption bands that confirm the presence of a primary amine (–NH_2_) group. The broad band observed at 3349 cm^−1^ is attributed to N–H stretching vibrations, indicating hydrogen bonding, while the band at 2962 cm^−1^ corresponds to aliphatic C–H stretching vibrations. The absorption at 1654 cm^−1^ is assigned to N–H bending (scissoring) vibrations, providing further evidence for the –NH_2_ functionality. Additionally, the band at 1573 cm^−1^ can be associated with C=C stretching vibrations or overlapping N–H vibrations. In the fingerprint region, the bands at 1483 and 1382 cm^−1^ are due to C–H bending vibrations, whereas the distinct band at 1322 cm^−1^ is characteristic of C–N stretching, confirming the attachment of the amine group to the carbon framework. Other bands observed at 1153, 994, 819, and 586 cm^−1^ are attributed to skeletal vibrations and out-of-plane C–H bending, which collectively support the proposed chemical structure of compound (A).

**Fig. 2:**
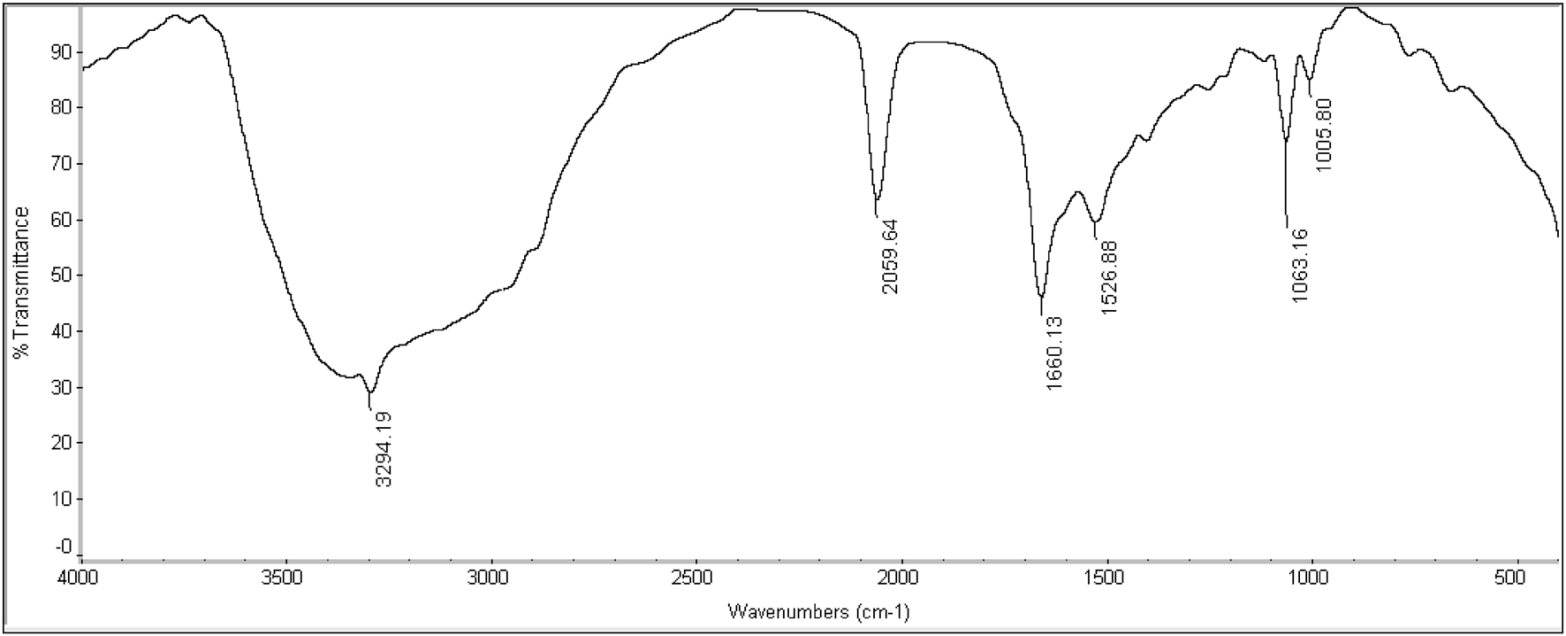
Infrared structure of the prepared compound (B).

Figure 2 is shown the FTIR spectrum of compound (B) displays several characteristic absorption bands that indicate specific functional groups within the molecule. The broad band observed at approximately 3294 cm^−1^ is attributed to O–H stretching vibrations, likely from an alcohol or phenol group, suggesting the presence of hydrogen bonding. The sharp band at 2059 cm^−1^ is characteristic of C≡N stretching vibrations, indicating the presence of a nitrile group. The strong absorption near 1660 cm^−1^ corresponds to C=O stretching vibrations, which may arise from a carbonyl group in a ketone, aldehyde, or amide. The bands at 1623 cm^−1^ and 1527 cm^−1^ can be assigned to C=C stretching vibrations of an aromatic ring or alkene, or possibly overlapping N–H bending vibrations if an amine or amide is present. In the fingerprint region, the band at 1005 cm^−1^ is associated with C–O stretching vibrations, further supporting the presence of an alcohol or ether group. Additional bands below 1000 cm^−1^ are due to skeletal vibrations and out-of-plane bending modes, which collectively confirm the proposed chemical structure of compound (B) as containing hydroxyl, nitrile, carbonyl, and unsaturated carbon–carbon bond functionalities.

**Fig. 3:**
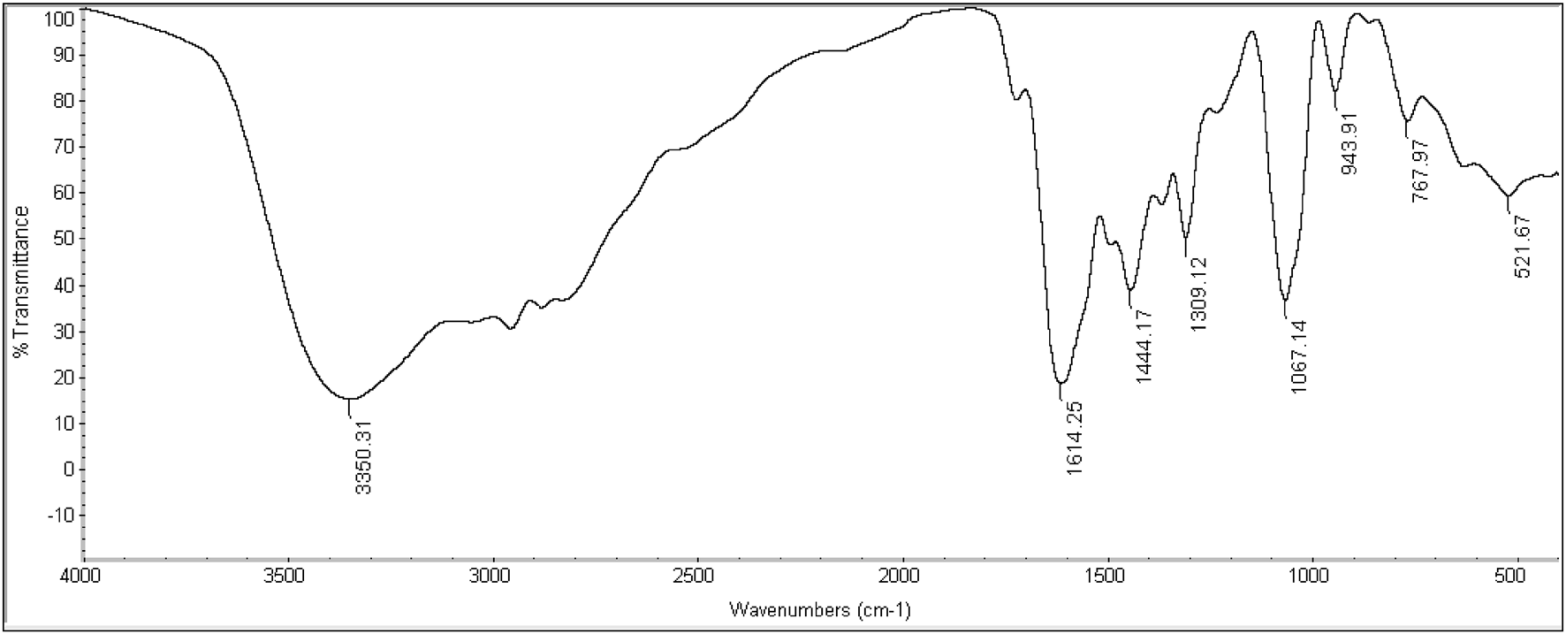
Infrared structure of the prepared compound (C).

Figure 3 is shown the FTIR spectrum of compound (C) shows characteristic absorption bands indicative of specific functional groups. The broad and strong band observed at 3500 cm^−1^ is attributed to O–H stretching vibrations, suggesting the presence of an alcohol or phenol group with extensive hydrogen bonding. The sharp band at 1614 cm^−1^ corresponds to C=C stretching vibrations, likely from an aromatic ring or an alkene. The band at 1444 cm^−1^ is due to C–H bending vibrations (scissoring or bending modes). The absorption at 1309 cm^−1^ may be assigned to C–N stretching vibrations or could overlap with C–O stretching, indicating possible amine or ether linkages. The strong band at 1067 cm^−1^ is characteristic of C–O stretching vibrations, further supporting the presence of an alcohol, ether, or ester group. In the fingerprint region, the bands at 944 cm^−1^, 768 cm^−1^, and 522 cm^−1^ are associated with skeletal vibrations and out-of-plane C–H bending modes, which are consistent with an aromatic substitution pattern or alkene geometry. These spectral features collectively suggest that compound (C) contains hydroxyl, unsaturated carbon–carbon bonds, and likely ether or amine functional groups within its structure.

### Quantum mechanical investigation

Molecular modeling and quantum mechanical computations on the synthesized compounds (A, B, and C) were performed with the aid of the ChemBioDraw Ultra software package. The investigation involved the computation of the geometrical structures, the optimized conformations of the molecules, and the distribution of the frontier molecular orbitals (FMOs), that is, the HOMO and LUMO densities, as shown in Tables 2, 3 and 4. The quantum chemical computations give a theoretical perspective regarding the molecular structure and their physicochemical properties. In that sense, the HOMO energy level denotes the donation ability of the molecule, while the LUMO energy level indicates the accepting ability of the molecules. The energy gap between the two energy levels (ΔE = E_LUMO_ − E_HOMO_) is regarded as a significant parameter in terms of the chemical reactivity, stability, and adsorption nature of the prepared compounds. As observed from the computed results, compounds A and B had low energy gap values (ΔE ≈ 1.10 eV), which implies good electronic mobility and high molecular polarizability. This makes the compounds able to easily donate electrons, thus improving their ability to engage in intermolecular interactions at the oil–water interface. Therefore, it means that these compounds have higher chances of being adsorbed at the interface, hence reducing the interfacial tension (IFT) between the oil and water phases. The phenomenon has a direct impact on the performance of surfactants during enhanced chemical oil recovery (cEOR). However, compound C exhibited a much higher energy gap value (ΔE = 2.477 eV), implying a higher level of stability but less chemical reactivity and interface activity. It can be explained by the specific structure and the electronic configuration of the substance, making it less flexible for the electron exchange reaction at the interface. In other words, this substance should act as an auxiliary or co-solvent rather than a primary surfactant.

**Table 2:** Geometric structures of prepared compound A.

Optimized structure and the frontier molecule orbital density distributions of prepared compound A		
	Optimized structure	
	HOMO	E_HOMO_ = − 4.920 eV
	LUMO	E_LUMO_ = − 3.817 eV

**Table 3:** Geometric structures of prepared compound B.

Optimized structure and the frontier molecule orbital density distributions of prepared compound B		
	Optimized structure	
	HOMO	E_HOMO_ = − 4.923 eV
	LUMO	E_LUMO_ = − 3.818 eV

**Table 4:** Geometric structures of prepared compound C.

Optimized structure and the frontier molecule orbital density distributions of prepared compound C		
	Optimized structure	
	HOMO	E_HOMO_ = − 4.698 eV
	LUMO	E_LUMO_ = − 2.221 eV

Moreover, the density distribution of frontier molecular orbitals indicated that the electrons were distributed in areas adjacent to heteroatoms, specifically N, O, and S. Such active sites allow effective interaction between the compound and water, crude oil components, and the rocks. The presence of unshared electrons leads to enhanced hydrogen bonding and polarity interactions between the compound and the rock surface. Consequently, such interaction contributes to wettability modification in favor of water-wet condition from the oil-wet state, facilitating oil displacement. In summary, the quantum mechanical analysis clearly supports the experimental results that were collected based on the surface tension, interfacial tension, and core flooding analyses. Indeed, there is a very strong correlation between structure and properties, wherein the electronic and geometrical structure of the synthesized molecules determines their surface activity.

### Surface and thermodynamic study

Surface active properties of the prepared compounds.

#### Critical micelle concentration (CMC) and surface tension at critical micelle concentration (γ_CMC_)

The self-assembly of a variety of amphiphile compounds in aqueous solution is essential to their application and is characterized by aggregation ability and aggregate morphology^53^. Parameters of micellization, such as critical micelle concentration (CMC), Maximum surface excess (Γmax), Minimum surface area (A_min_), and various thermodynamic values etc. play important roles in different applications.

**Fig. 4:**
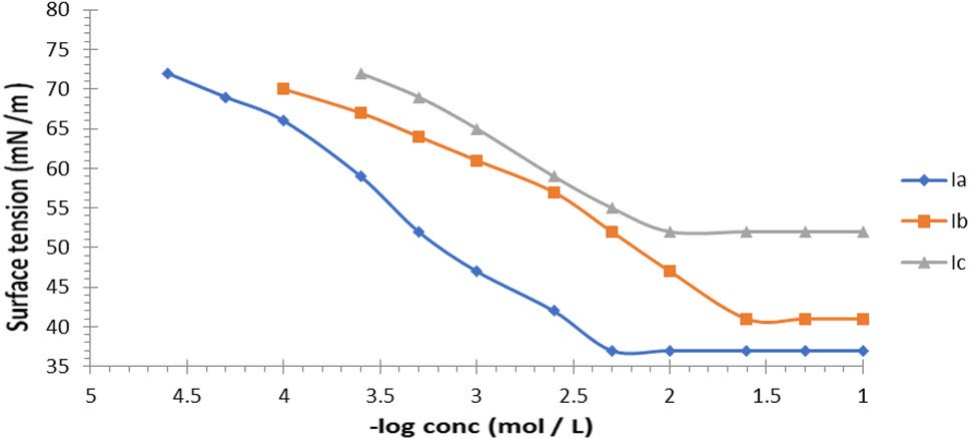
Variation of the surface tension with − log concentrations for the synthesized compounds (Ia, Ib, and Ic) at 25 °C.

The surface parameters of the synthesized compounds depended on the functional group of the synthesized compounds. The CMC values of Ia, Ib, and Ic were determined and corresponded to the intersection points in the difference in surface tension of the synthesized compounds versus -log molar concentration (− log c) (Fig. 4). The calculated CMC values at 25 °C were summarized in Table 5. This was ascribed to the increase in the solution hydrophobicity, which destroyed the water structure and enhanced the free energy of the acquired system, triggering the aggregation of the amphiphile molecules into a micelle structure or migration to the surface of the solution. This behavior resulted in the relaxation in the system free energy, consequently increasing the hydrophobic chain length inducing a decrease in the CMC values^54–56^. Generally, the synthesized compounds show good surface-active and self-aggregation properties in aqueous solutions. Therefore, it can be concluded that the compound Ia revealed the optimum value of CMC than Ib and Ic.

**Table 5:** Critical micelle concentration (CMC) and surface parameters of organic compounds at 25 °C.

Surfactants	CMC × 10^–2^ (mol/l)	γ_CMC_ (mN/m)	Π_CMC_ (mN/m)	Γ_max_ × 10^–11^ (mol/cm^2^)	A_min_ (nm^2^)	ΔG_ads_ (kj/mol)	ΔG_mic_ (kj/mol)
Ia_(bis N)_	0.0723	38.28	33.72	9.58	1.73	− 18.0	− 17.92
Ib_(bis 2)_	0.0943	52.88	19.12	9.67	1.72	− 17.3	− 17.26
Ic_(bis 1)_	0.117	55.93	16.07	7.26	2.29	− 16.7	− 16.73

In Table 5, the surface tension values at the respective CMCs (γCMC) for the prepared compounds Ia, Ib, and Ic at 25 °C were determined. The results revealed the same behavior as shown in CMC. Therefore, it can be concluded that the Compound Ia possess lower surface tension (γCMC) value than the other ones.

#### Effectiveness (π_cmc_)

The π_cmc_ values of the Ia, Ib, and Ic were determined (Table 5). The π_cmc_ values indicate the degree of packing of molecules at the solution surface. Specifically, high values of π_cmc_ refer to the formation of a compact layer at the interface, whereas low values indicate loose packing of the molecules at the interface^57^.

#### Maximum surface excess (Γ_max_) and Minimum surface area (A_min_)

The Γ_max_ and A_min_ values of the aqueous solutions of Ia, Ib, and Ic were calculated using the Gibbs adsorption Eqs. (2) and (3), respectively (Table 5). The Γ_max_ values indicate the amount of accumulated Ia, Ib, and Ic molecules at the interface, whereas A_min_ refers to their ordering density at the interface. The A_min_ values of the synthesized compounds were determined. In addition, the corresponding Γ_max_ values were calculated. Value of Γ_max_ indicates that the slope of the surface tension plot (*δ*γ/*δ log c*) effect on the value of maximum surface excess. Notably, the higher the surface excess concentration value, the lower the surface area value^58,59^.

#### Thermodynamic properties of the synthesized compounds

Standard free energies of micellization and adsorption (ΔG^o^_mic_, ΔG^o^_ads_).

The thermodynamic behavior of the synthesized compounds Ia, Ib, and Ic was analyzed using the Gibbs Eqs. (4) and (5)^60–64^. The determined thermodynamic parameters are summarized in Table 5. It is noteworthy that both the change in the micellization free energy ((ΔG^o^_mic_) and adsorption (ΔG^o^_ads_) of the synthesized Ia, Ib, and Ic compounds were negative, indicating that the adsorption and micellization processes were spontaneous^65^. This minimized the increase in the solution energy resulting from the dissolution of the amphiphilic compounds in the aqueous system. The data shown in Table 5 suggest that ΔG^o^_ads_ for the synthesized compounds was more negative than the corresponding ΔG^o^_mic_. This implied that in aqueous solution, synthesized Ia, Ib, and Ic first adsorbed at the solution interface until reaching maximum saturation, and subsequently aggregated, forming a micelle structure.

#### Interfacial tension measurement

Interfacial tension is the force that acts at the interface between two immiscible liquids. This force is caused by the imbalance of intermolecular forces at the interface. The interfacial tension between crude oil and distillate water was 30 mN/m while between crude oil and formation water was 27 mN/m. When we measured the interfacial tension between crude oil and compound Ia (bis N) slug at concentration (0.1% w/v). The interfacial tension of sample Ia was estimated three times (2 min taken between each reading), we found more reduction in IFT and became 5 mN/m This indicated that compound slug can strongly reduce the IFT between trapped residual oil and formation water, enhancing the mobilization of immobile oil and improving the oil recovery.

### Experimental results of reservoir core plugs

#### Wettability

The test results (Tables 6 and 7) showed that the tested samples have oil-wetting tendencies, where the water indixes were 0.03, 0.00, and 0.031 for samples (# 1), (# 2), and (# 3) respectively, on the other hand, the oil indixes were 0.61, 0.86, and 0.87 for samples (# 1), (# 2), and (# 3) respectively. The results may indicate that the residual oil saturation results from the oil–water interfacial tension value and wetting characteristics.

**Table 6:** The indexes of water wet and oil wet of the three core samples.

Sample No	Water wet index			Oil wet index		
	Fw. Imbibed statically (CC)	Fw. Imbibed dynamically (CC)	Water wet index	Oil imbibed statically (CC)	Oil imbibed dynamically (CC)	Oil wet index
# 1	0.10	3.80	0.03	2.20	1.40	0.61
# 2	0.00	4.85	0.00	5.10	0.80	0.86
# 3	0.15	4.75	0.031	3.75	0.55	0.87

**Table 7:** The final results of the three core samples wettability.

Sample No	Water wet index	Oil wet index	Wet index	Final results
# 1	0.03	0.61	− 0.59	Oil wet
# 2	0.00	0.86	− 0.86	Oil wet
# 3	0.03	0.87	− 0.84	Oil Wet

#### Core flooding

##### Secondary water flooding phase

**Fig. 5:**
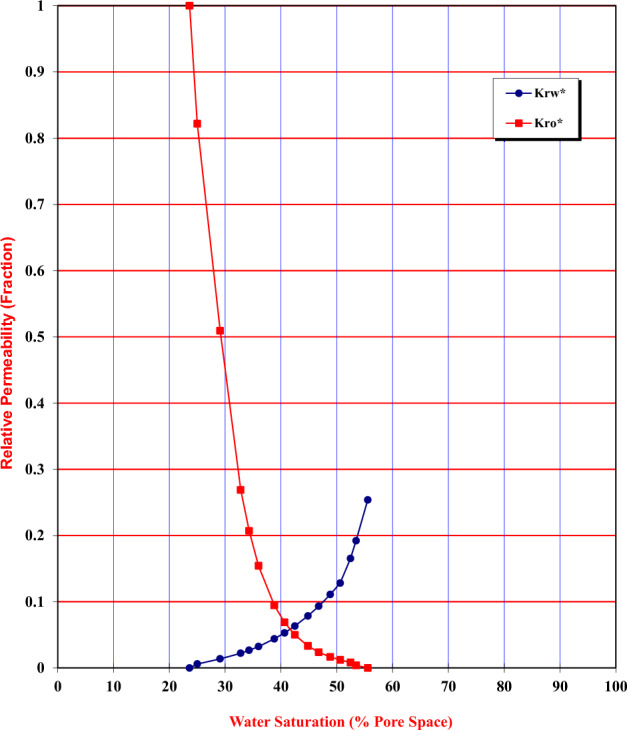
Relative permeability versus average water saturation of sample # 1.

**Fig. 6:**
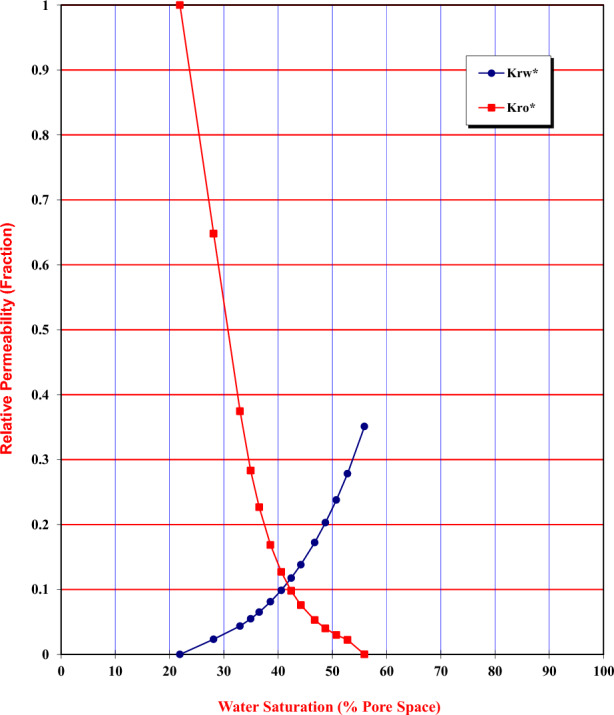
Relative permeability versus average water saturation of sample # 2.

**Fig. 7:**
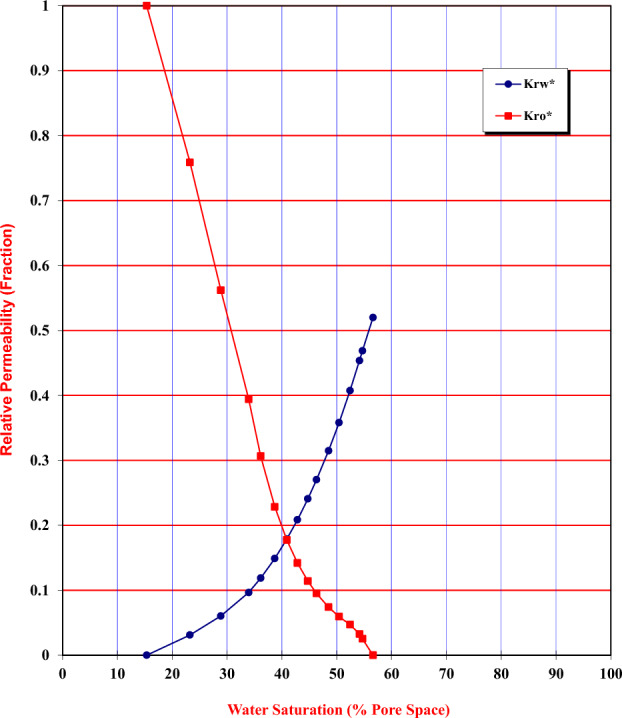
Relative permeability versus average water saturation of sample # 3.

The petrophysical properties of the tested plugs and flooding conditions are displayed (Tables 1 and 8). The plugs of interest are similar in terms of lithology, where they are sandstone, greenish gray color, fine grains, cemented, and glauconite. The results of relative permeability (Figs. 5, 6 and 7) at reservoir conditions (secondary flooding), displayed that the tested samples (# 1, # 2 & # 3) have wetting characteristics to oil, where the cross-over point is lesser than 50% water saturation as a rule of thumb and this agrees with the wettability results. The results of secondary recovery (Table 8) at reservoir conditions, displayed that the residual oil that was left in the three tested core samples was 58.17% OOIP in the case of core sample (# 1), 56.43% OOIP in the case of core sample (# 2) and 51.25% OOIP in the case of core sample (# 3). The significant residual oil in the three core samples suggested some factors hindering oil recovery, such as a high interfacial tension between the oil and injected water, along with the oil-wet nature of the samples.

**Table 8:** The different parameters of the tested plugs and flooding conditions.

Sample No	S_wi_ (% P.V.)	S_wi_, CC	OOIP, CC	OOIP (% P.V.)	K_o_ @ S_wi_ (mD)	S_or_ (% PV)	S_or_, CC	S_or_ (%), OOIP	K_w_@ S_or_ (mD)
# 1	23.62	3.72	12.05	76.38	64.50	44.44	7.01	58.17	16.37
# 2	21.90	3.51	12.51	78.10	76.25	44.07	7.06	56.43	26.76
# 3	15.34	2.38	13.15	84.66	44.99	43.37	6.74	51.25	23.39
**Flooding conditions**									
Flooding temperature, °C				60					
Confining pressure, psi				2700					
Brine salinity, ppm				95,000					

S_wi_: Irreducible water saturation (% pore volume), k_o_ @S_wi_: Oil permeability at irreducible water saturation, S_or_ (% P.V.): Residual oil saturation (% pore volume), S_or_ (% OOIP): Residual Oil Saturation (% OOIP), K_w_ @S_or_: Water permeability at residual oil saturation

##### Tertiary chemical flooding (EOR)

**Fig. 8:**
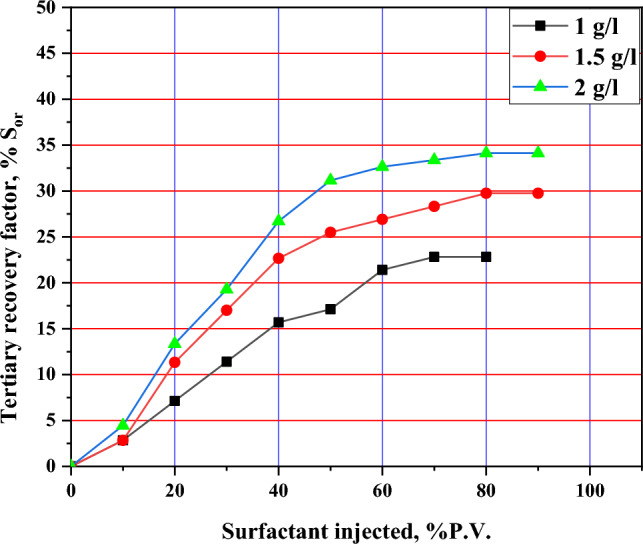
Oil recovery percentage (%) S_or_ using different concentrations of surfactant solution.

**Fig. 9:**
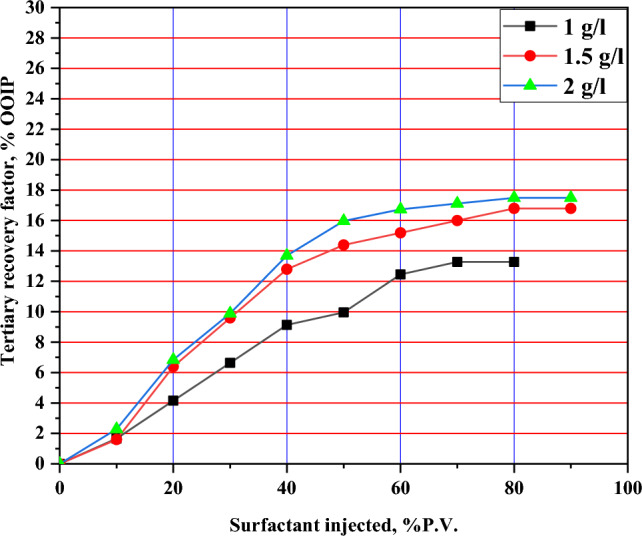
Oil recovery percentage (%) OOIP using different concentrations of surfactant solution.

Following the core water flooding the chemical flooding using compound Ia with three different concentrations (1 g/l, 1.5 g/l and 2 g/l) for samples (# 1), (# 2), and (# 3). The results are shown (Tables 9, 10 and 11) and represented graphically (Figs, 8 and 9). They indicate positive economics for enhanced oil recovery through different concentration of this new surfactant where they achieve a recovery factor of 22.82%S_or_ in the case of (1 g/l), 29.75%S_or_ in the case of (1.5 g/l) and 34.12%S_or_ in the case of (2 g/l) from the remaining oil. These results support the results of ITF and wettability tests (Figs. 8 and 9).

**Table 9:** Cumulative oil recovery as a function of pore volume injected in the case of sample (# 1) (1 g/l).

Surfactant injected, % P.V	Tertiary oil recovery, %, s_or_	Tertiary oil recovery, % OOIP
0	0	0
6.02	2.85	1.66
11.10	7.13	4.15
17.44	11.41	6.64
23.78	15.69	9.13
27.58	17.12	9.96
32.02	21.40	12.45
36.46	22.82	13.28
42.17	22.82	13.28

**Table 10:** Cumulative oil recovery as a function of pore volume injected in the case of sample (# 2) (1.5 g/l).

Slug injected, % P.V	Tertiary oil recovery, %, s_or_	Tertiary oil recovery, % OOIP
0	0	0
5.93	2.83	1.60
10.92	11.33	6.39
17.17	17.00	9.59
23.41	22.66	12.79
27.15	25.50	14.39
31.52	26.91	15.19
35.89	28.33	15.99
41.51	29.75	16.79
56.49	29.75	16.79

**Table 11:** Cumulative oil recovery as a function of pore volume injected in the case of sample (# 3) (2 g/l).

Slug injected, % P.V	Tertiary oil recovery, %, s_or_	Tertiary oil recovery, % OOIP
0	0	0
6.12	4.45	2.28
11.27	13.35	6.84
17.71	19.29	9.89
24.15	26.71	13.69
28.01	31.16	15.97
32.52	32.64	16.73
37.03	33.38	17.11
42.82	34.12	17.49
58.27	34.12	17.49

##### Quantum chemical insights into EOR efficacy

The quantum chemical parameters, particularly the narrow energy gap (ΔE) between the highest occupied (E_HOMO_) and lowest unoccupied (E_LUMO_) molecular orbitals (Table 12) provide a fundamental theoretical basis for understanding their efficacy in reducing interfacial tension (IFT) and enhancing oil recovery (EOR). Compounds A and B, with small ΔE values of approximately 1.10 eV, exhibit high polarizability and electronic mobility, which facilitate strong adsorption at the oil–water interface, significantly lowering IFT by disrupting cohesive forces. This direct correlation between a low ΔE and increased surface activity explains their superior performance as surfactants in EOR, where minimizing IFT is critical for increasing the capillary number and mobilizing trapped oil. In contrast, the larger ΔE of Compound C (2.477 eV) suggests lower interfacial activity, aligning with its potential role as a co-solvent rather than a primary surfactant. Thus, these quantum descriptors theoretically validate the structure–function relationship, where electronic properties govern interfacial behavior, enabling the rational design of chemical formulations for efficient EOR applications.

**Table 12:** Quantum chemical parameters.

Code of prepared compounds	E_LUMO_ (eV)	E_HOMO_ (eV)	ΔE = (E_HOMO_ − E_LUMO_) (eV)	Absolute ΔE
A	− 3.817	− 4.920	−1.103	1.103
B	− 3.818	− 4.923	− 1.105	1.105
C	− 2.221	− 4.698	− 2.477	2.477

## Conclusion

In this study, three organic derivatives (Ia, Ib, and Ic) were synthesized and evaluated for their potential in chemical enhanced oil recovery (cEOR) applications. The performance of these compounds was assessed through a combination of surface activity measurements, petrophysical characterization, and core-flooding experiments. Based on the experimental and theoretical results, the following conclusions can be drawn:

- **Synthesis and Characterization:** FTIR spectroscopy successfully confirmed the structural integrity of the synthesized compounds. For compound Ia, the presence of specific amine functional groups facilitated optimal orientation and adsorption at the oil–water interface.
- **Surface and Interfacial Activity:** Among the tested derivatives, compound Ia demonstrated the highest efficiency in reducing surface and interfacial tension. The experimental findings were supported by quantum chemical calculations, where the narrow energy gap (ΔE) of compound Ia indicated higher reactivity and interfacial adsorption compared to Ib and Ic.
- **Incremental Oil Recovery:** Core-flooding experiments under reservoir conditions confirmed the efficacy of compound Ia. Incremental oil recovery factors reached 22.82%, 29.75%, and 34.12% of residual oil (S_or_) at concentrations of 1 g/L, 1.5 g/L, and 2 g/L, respectively.
- **Structure–Property Correlation:** A strong correlation was observed between the theoretical descriptors (DFT) and the experimental IFT results, validating the selection of compound Ia as a promising candidate for chemical flooding.

## Limitations and future work

While the results demonstrate the potential of compound Ia, it is important to acknowledge that this study was conducted under specific laboratory-controlled reservoir conditions. The long-term thermal stability of these compounds over several months and their adsorption behavior on different rock mineralogies (e.g., carbonate vs. sandstone) remain areas for further investigation. Future research will focus on the environmental biodegradability of these derivatives and their performance in pilot-scale field applications to ensure long-term economic and ecological viability.

## Data Availability

The raw data used and analyzed during the current study is available from the corresponding author upon reasonable request.

## Abbreviations

BV: Bulk volume
cEOR: Chemical enhanced oil recovery
CMC: Critical micelle concentration
CO₂: Carbon dioxide
DFT: Density functional theory
EGR: Enhanced gas recovery
EOR: Enhanced oil recovery
FTIR: Fourier transform infrared spectroscopy
GV: Grain volume
HTHS: High temperature high salinity
IFT: Interfacial tension
IOR: Improved oil recovery
K: Permeability
mN/m: Milli-Newton per meter
N₂: Nitrogen
NMR: Nuclear magnetic resonance
OOIP: Original oil in place
Ppm: Parts per million
PV: Pore volume
S_or_: Residual oil saturation
ST: Surface tension
S_wi_: Initial water saturation
Ø: Porosity
